# Predictors of Progressive Fibrosing Interstitial Lung Diseases and Survival in Fibrosing Interstitial Lung Disease-Related Usual Interstitial Pneumonia

**DOI:** 10.3390/medicina62010206

**Published:** 2026-01-19

**Authors:** Hongyan Fu, Xiao Li, Hongyang Shi, Jie Zhang, Ming Zhang

**Affiliations:** Department of Pulmonary and Critical Care Medicine, The Second Affiliated Hospital of Xi’an Jiaotong University, Xi’an 710004, China; 18710724031@163.com (X.L.); shihy2003@126.com (H.S.); zhangjie@xjtu.edu.cn (J.Z.); zhangmingdr@163.com (M.Z.)

**Keywords:** usual interstitial pneumonia, progressive fibrosing interstitial lung diseases, fibrosing interstitial lung disease, quality of life

## Abstract

*Background and Objectives:* Usual interstitial pneumonia (UIP) is associated with progressive fibrosing interstitial lung diseases (PF-ILD) and poor survival in patients with fibrosing interstitial lung disease (FILD). We aimed to investigate the predictors of PF-ILD and survival in patients with FILD-UIP. *Materials and Methods:* This retrospective study was conducted at a single, tertiary hospital in China. Patients underwent routine follow-up visits every 3 to 6 months according to standard operating procedures (SOPs). Patients with FILD-UIP were further stratified using the proposed PF-ILD criteria. *Results*: This retrospective study enrolled 150 patients with FILD-UIP between October 2020 and June 2025, with 117 patients completing follow-up for more than 12 months. FILD-UIP was categorized as idiopathic pulmonary fibrosis (IPF) (*n* = 67) and non-IPF-UIP (*n* = 50), which included connective tissue disease-associated UIP (*n* = 29), hypersensitivity pneumonitis-associated UIP (*n* = 7), and interstitial pneumonia with autoimmune features-associated UIP (*n* = 14). During the follow-up period, 32 (47.8%) patients with IPF and 19 (38.0%) non-IPF-UIP experienced PF-ILD. Pulmonary hypertension (PH) and predicted percentage of forced vital capacity (FVC%pred) were dependent risk factors for PF-ILD in patients with FILD-UIP, non-IPF-UIP, and IPF. King’s Brief Interstitial Lung Disease (KBILD) is a dependent risk factor for PF-ILD in patients with FILD-UIP and IPF. PF-ILD is similarly associated with poor survival in patients with FILD-UIP, non-IPF-UIP, and IPF. *Conclusions*: Baseline disease severity is closely associated with the incidence of PF-ILD, with all forms of FILD-UIP at risk of PF-ILD and showing similar outcomes to IPF-UIP/PF-ILD.

## 1. Introduction

Fibrosing interstitial lung diseases (FILD) are a heterogeneous group of disorders with a variable and enigmatic course [1]. Idiopathic pulmonary fibrosis (IPF) is a type of progressive FILD characterized by a radiological and/or histopathological pattern of usual interstitial pneumonia (UIP). However, patients with non-IPF-UIP, including connective tissue disease-associated UIP (CTD-UIP), hypersensitivity pneumonitis-associated UIP (HP-UIP), and interstitial pneumonia with autoimmune features-associated UIP (IPAF-UIP), are also at high risk for developing progressive fibrosing phenotype, similar to IPF, despite receiving standard treatment during the disease course [2]. A progressive fibrosing phenotype develops in approximately 39.21% of patients with FILD overall group [3]. A retrospective study found that up to 48% of patients with non-IPF FILD have a progressive fibrosing phenotype [4]. The progression of FILD, characterized by worsening dyspnea and quality of life (QOL), deterioration of lung function, increased extent of pulmonary fibrosis on high-resolution computed tomography (HRCT), and a detrimental prognosis, has recently been described as progressive pulmonary fibrosis (PPF), also known as progressive fibrosing interstitial lung disease (PF-ILD). PF-ILD places a significant burden on patients, impairing both physical and emotional well-being and leading to reduced QOL [5].

In clinical practice, pulmonary function tests (PFTs) serve as a standard tool to measure disease progression, while HRCT is used to detect changes in the extent of pulmonary fibrosis [6]. However, monitoring disease progression also includes patient-reported outcomes and exercise capacity assessments. By gathering responses directly from patients, these measures inherently include patients’ values and judgments and can be evaluated using QOL questionnaires [7]. Rajala et al. [8] and Lee et al. [9] reported that in IPF patients, an increasing mMRC score is associated with impaired quality of life and a higher symptom burden, while depression and anxiety are highly prevalent and significantly worsen patients’ QOL. Sharp et al. [10] reported that King’s Brief Interstitial Lung Disease (KBILD) has prognostic power equivalent to pulmonary physiology and exercise testing in ILD at a single point in time. The 6-min walk test (6MWT) is a practical, inexpensive, and reliable tool for assessing exercise capacity, prognosis, and treatment response across a wide range of respiratory diseases [11].

Predictors of PF-ILD in patients with FILD have been investigated in several studies. Risk factors such as the neutrophil-to-lymphocyte ratio and a UIP pattern on HRCT have been identified [3], with lower forced vital capacity (FVC) being an independent predictor for PF-ILD in non-IPF FILD [6]. The oxygenation status during the 6MWT may also provide prognostic value in defining disease progression in FILD [12]. However, data on the frequency and prognosis of PF-ILD specifically in patients with FILD-UIP remain limited [13,14,15]. Therefore, we performed a retrospective cohort study of patients with FILD-UIP (including both IPF and non-IPF-UIP to explore potential factors associated with PF-ILD and mortality.

## 2. Methods

### 2.1. Study Population

**Figure 1:**
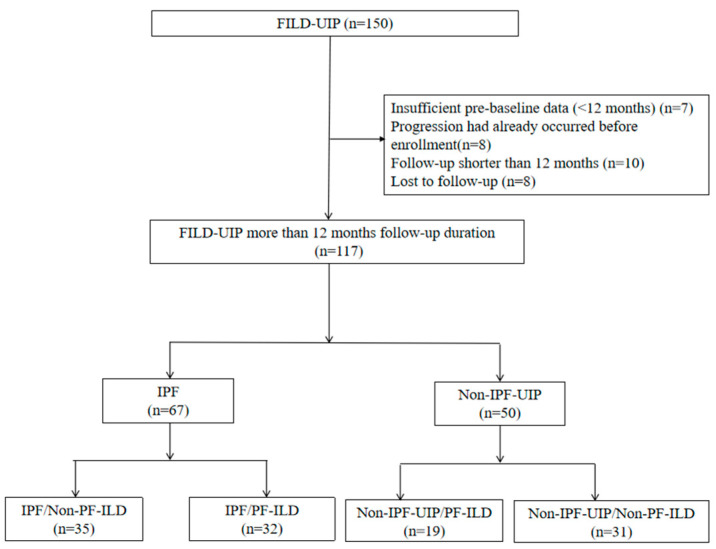
Study flow chart. FILD, fibrosing interstitial lung disease; IPF, idiopathic pulmonary fibrosis; PF-ILD, progressive fibrosing interstitial lung disease; UIP, usual interstitial pneumonia.

This single-center, retrospective study was conducted at the Second Affiliated Hospital of Xi’an Jiaotong University (Xi’an, China). Patients hospitalized between October 2020 and June 2025 with FILD-UIP were enrolled following multidisciplinary discussion involving pulmonologists, radiologists, pathologists, and rheumatologists. Participants were categorized as having IPF-UIP or non-IPF-UIP, the latter including CTD-UIP, HP-UIP, and IPAF-UIP. All enrolled patients were in a stable disease state without evidence of PF-ILD in the 12 months prior to baseline. The INBUILD criteria were applied using all available data from the 12 months preceding the baseline date to confirm progressive disease at study entry. A total of 150 patients diagnosed with FILD-UIP were initially enrolled. After excluding 33 patients—7 due to insufficient pre-baseline data (<12 months) to confirm progression status at baseline, 8 due to disease progression had already occurred before enrollment, 10 due to a follow-up period shorter than 12 months, and 8 due to loss to follow-up—117 patients who completed more than 12 months of follow-up were included in the final analysis (Figure 1). The diagnosis of UIP at our department is made through a rigorous multidisciplinary process involving two expert thoracic radiologists. The diagnosis of IPF was based on the Official American Thoracic Society (ATS)/European Respiratory Society (ERS)/Japanese Respiratory Society (JRS)/Latin American Thoracic Association (ALAT) guidelines for IPF [16]. The diagnosis of CTD-UIP, including UIP associated with rheumatoid arthritis (RA), systemic sclerosis (SSc), and polymyositis/dermatomyositis, was based on the published guideline [17,18,19,20]. The diagnosis of HP met the diagnostic criteria of the ATS/JRS/ALAT clinical practice guidelines for HP [21]. IPAF was diagnosed using the officially accepted ERS/ATS research statement for IPAF, incorporating both clinical and serological domains [22]. The diagnosis of pulmonary hypertension (PH) was based on transthoracic echocardiography. According to the ESC/ERS guidelines for the diagnosis and treatment of pulmonary hypertension, a peak tricuspid regurgitation velocity (TRV) >2.8 m/s is recommended as the threshold for echocardiographic probability of PH. Systolic PAP is estimated based on the peak TRV and right atrial pressure (RAP) as described by the simplified Bernoulli equation. sPAP = RVSP = 4 × TRV^2^ + RAP. According to this equation, an sPAP greater than 35 mmHg indicates the possibility of PH [23,24,25,26].

### 2.2. Definition of PF-ILD

Patients with PF-ILD were classified based on the INBUILD trial criteria [27], defined as meeting at least one of the following criteria within the 24 months, despite standard treatment: a relative decline in the forced vital capacity of at least 10% of the predicted value; a relative decline in the forced vital capacity of 5% to less than 10% of the predicted value and worsening of respiratory symptoms or an increased extent of fibrosis on HRCT; or worsening of respiratory symptoms and an increased extent of fibrosis. Patients were excluded if they had conditions that could have contributed to disease progression, such as pulmonary embolism, decompensated heart failure, or pneumothorax.

### 2.3. Data Collection

Clinical and survival data were obtained from hospital records, outpatient follow-up records, hospitalization records, and telephone communications. Collected variables included demographic data; QOLs (KBILD, modified Medical Research Council [mMRC], Hospital Anxiety and Depression Scale [HADS], Leicester Cough Questionnaire [LCQ]); comorbidities; functional data (FVC% predicted, carbon monoxide diffusing capacity [DLCO]% predicted, 6-min walk test [6MWT]); laboratory data; HRCT; and pharmacologic treatments. Patients underwent routine follow-up visits every 3 to 6 months according to standard operating procedures (SOPs). Once participants were suspected of having PF-ILD, the diagnosis was confirmed by a multidisciplinary team. While partial follow-up data were obtained from hospitalization records, we carefully distinguished between acute events (e.g., pneumonia or acute exacerbation) and true PF-ILD. Overall survival time was defined as the duration from the date of diagnosis to the date of death from any cause or the date of the last follow-up.

### 2.4. Statistical Analysis

Continuous variables with a normal distribution are presented as means with standard deviations (SD), while those without a normal distribution are presented as medians with interquartile ranges (IQR). Categorical variables were expressed as numbers and percentages. Continuous variables were analyzed using the unpaired *t*-test for normally distributed data or the Mann–Whitney U test for non-normally distributed data or when *t*-test assumptions were violated. Categorical variables were compared using the chi-square test (for expected cell frequencies ≥5) or Fisher’s exact test (for any expected frequency <5). The incidence of PF-ILD was calculated by dividing the number of patients developing PF-ILD by the total number of patient years. Risk factors for PF-ILD were analyzed using logistic regression analysis, and continuous variables were converted into dichotomous variables using the median cut-off. Survival curves were generated using the Kaplan–Meier method, and prognostic factors for mortality were identified through Cox proportional hazards regression. For the non-IPF subgroup, where the number of death events was limited (*n* = 12), multivariable adjustment was constrained by statistical power. Analyses are primarily based on univariable Cox models. A *p*-value < 0.05 was considered statistically significant.

## 3. Results

### 3.1. Characteristics of Patients with FILD-UIP

**Figure 2:**
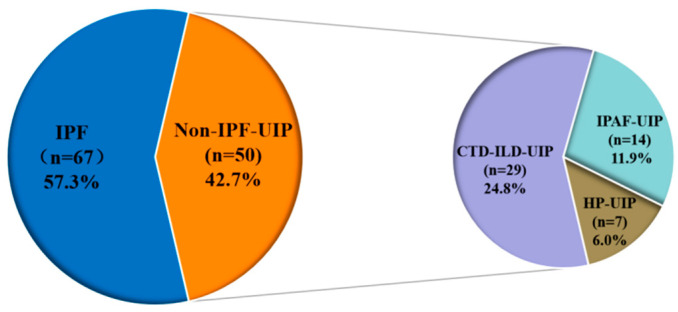
Distribution of different ILDs in the FILD. CTD, connective tissue disease; HP, hypersensitivity pneumonitis; IPAF, interstitial pneumonia with autoimmune features; IPF, idiopathic pulmonary fibrosis; UIP, usual interstitial pneumonia.

The clinical characteristics of patients with FILD characterized by UIP on HRCT (*n* = 117) are summarized in Table 1. A total of 67 patients with IPF and 50 patients with non-IPF-UIP were included. Of the non-IPF-UIP cases among FILD patients, the most frequent diagnoses were CTD-UIP (*n* = 29, 24.8%), IPAF-UIP (*n* = 14, 11.9%), and HP-UIP (*n* = 7, 6.0%) (Figure 2). Among 29 patients with CTD-ILD, RA-UIP was the most frequent type (*n* = 21, 72.4%), followed by SSc-UIP (*n* = 5, 17.2%), and myositis-UIP (*n* = 3, 10.4%). Among patients with IPF, 47.8% (*n* = 32) of patients with IPF met the PF-ILD definition during an average follow-up of 37.4 ± 14.6 months, whereas 38.0% (*n* = 19) of patients with non-IPF-UIP met the PF-ILD definition during an average follow-up of 41.1 ± 13.0 months. Among 19 patients with non-IPF-UIP/PF-ILD, RA-UIP was the most frequent (*n* = 6, 31.6%), followed by HP-UIP (*n* = 5, 26.3%), IPAF-UIP (*n* = 5, 26.3%), SSc (*n* = 2, 10.5%), and myositis (*n* = 1, 5.3%). Compared to patients with non-IPF-UIP, patients with IPF were older and had a higher proportion of males and antifibrotic treatment at baseline (*p* = 0.019, *p* = 0.008, and *p* < 0.001, respectively), and a lower proportion of steroid and immunosuppressive therapy (all *p* < 0.001). Baseline characteristics of patients with different Non-IPF-UIP are provided in the Supplementary Table S1. Relative to patients with CTD-UIP or HP-UIP, those with IPAF-UIP had a significantly higher proportion of males (*p* = 0.008). Furthermore, compared with patients in the CTD-UIP or IPAF-UIP subgroups, those with HP-UIP were less likely to have received immunosuppressive therapy (*p* = 0.008).

### 3.2. Patient Characteristics for IPF and Non-IPF-UIP Stratified by the PF-ILD Status

A comparison of patient characteristics between the IPF and non-IPF-UIP groups stratified by PF-ILD status is summarized in Table 2. IPF/PF-ILD patients had a lower body mass index (BMI) than that of IPF/non-PF-ILD patients (*p* = 0.005); however, no significant difference was observed between non-IPF-UIP/PF-ILD patients and non-IPF-UIP/non-PF-ILD patients. Additionally, IPF/PF-ILD patients had a higher rate of PH than that of IPF/non-PF-ILD patients (*p* < 0.001), with significant differences also noted between patients with non-IPF-UIP/PF-ILD and non-IPF-UIP/non-PF-ILD patients (*p* < 0.001).

### 3.3. QOL and Functional Parameters for IPF and Non-IPF-UIP Stratified by the PF-ILD Status

A comparison of QOL and functional parameters for patients with IPF and non-IPF-UIP stratified by PF-ILD status is summarized in Table 3. In IPF patients, those with PF-ILD showed significant differences in the KBILD score, mMRC score, FVC%pred, DLCO%pred, PaO_2_/FiO_2_ ratio, 6-min walk distance (6MWD), baseline SpO_2_ at 6MWT, and post-exercise SpO_2_ at 6MWT compared to the IPF/non-PF-ILD patients (*p* < 0.001, *p* < 0.001, *p* < 0.001, *p* = 0.001, *p* = 0.012, *p* = 0.001, *p* = 0.005, and *p* = 0.038, respectively). Similarly, among non-IPF-UIP patients, those with PF-ILD differed significantly in these parameters compared to non-PF-ILD patients (*p* = 0.011, *p* = 0.004, *p* = 0.017, *p* = 0.029, *p* = 0.039, *p* = 0.001, *p* = 0.045, and *p* = 0.001, respectively).

### 3.4. Laboratory, Treatment, and Survival Parameters for IPF and Non-IPF-UIP Stratified by the PF-ILD Status

Comparisons of laboratory, treatment, and survival parameters between patients with IPF and non-IPF-UIP, stratified by PF-ILD status, are summarized in Table 4. Lactate dehydrogenase (LDH) levels were significantly higher in IPF/PF-ILD patients than in the IPF/non-PF-ILD patients (*p* = 0.029). Mortality was significantly greater in IPF/PF-ILD patients than in the IPF/non-PF-ILD patients (68.8% vs. 8.6%; *p* < 0.001), and non-IPF-UIP/PF-ILD was similarly associated with poor survival (52.6% vs. 6.5%; *p* < 0.001).

### 3.5. Predictive Factors of PF-ILD

Predictive factors for FILD-UIP/PF-ILD, non-IPF-UIP/PF-ILD, and IPF/PF-ILD are presented in Table 5A, 5B, and 5C, respectively. Multivariate analysis revealed that, in patients with FILD-UIP, PH (OR, 6.056; 95% CI: 1.664–22.047, *p* = 0.006), KBILD (OR, 4.529; 95% CI: 1.592–12.884, *p* = 0.005), and FVC%pred (OR, 11.455; 95% CI: 2.144–10.690, *p* = 0.037) were significant independent risk factors for PF-ILD (Table 5A). In patients with non-IPF-UIP, PH (OR, 11.1123; 95% CI: 1.833–67.491, *p* = 0.009) and FVC%pred (OR, 4.762; 95% CI: 1.074–21.103, *p* = 0.04) were significant independent risk factors for PF-ILD (Table 5B). Similarly, in patients with IPF, PH (OR: 8.022, 95% CI: 1.104–58.282, *p* = 0.04), KBILD (OR: 16.297, 95% CI: 3.854–68.918, *p* < 0.001), and FVC%pred (OR: 5.478, 95% CI: 1.195–25.127, *p* = 0.029) were independent risk factors for PF-ILD in patients with IPF (Table 5C).

### 3.6. Predictive Characteristics Associated with Survival

**Figure 3:**
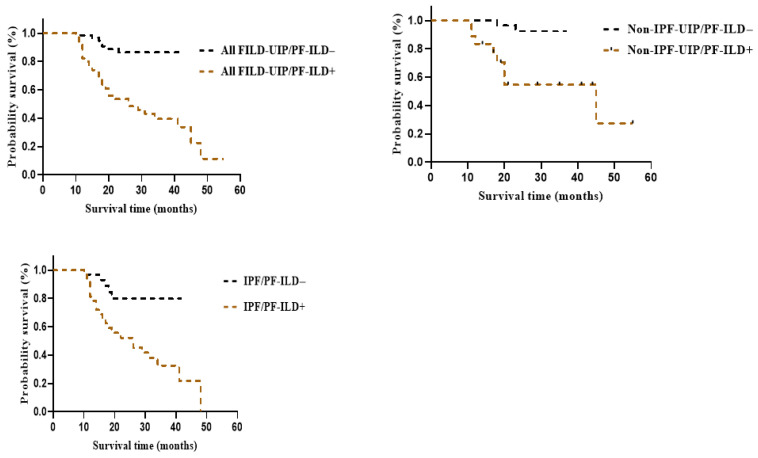
Kaplan–Meier curves for the transplant-free survival. Survival with or without PF-ILD. FILD, fibrosing interstitial lung disease; IPF, idiopathic pulmonary fibrosis; PF-ILD, progressive fibrosing interstitial lung disease; UIP, usual interstitial pneumonia.

**Figure 4:**
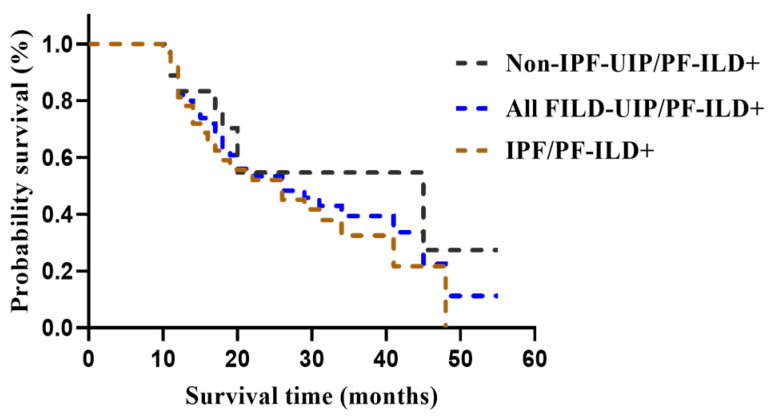
Kaplan–Meier curves for the transplant-free survival. FILD-UIP/PF-ILD, non-IPF-UIP/PF-ILD, and IPF/PF-ILD were associated with similar poor survival. FILD, fibrosing interstitial lung disease; IPF, idiopathic pulmonary fibrosis; PF-ILD, progressive fibrosing interstitial lung disease; UIP, usual interstitial pneumonia.

Multivariate Cox regression analysis revealed that PF-ILD was associated with early mortality in the FILD-UIP, non-IPF-UIP, and IPF groups (HR: 6.324, 95% CI: 2.442–16.379, *p* < 0.001; HR: 14.270; 95% CI: 1.640–124.150, *p* = 0.002; and HR: 4.939, 95% CI: 1.313–18.577, *p* = 0.018, respectively) (Supplementary Table S2). Kaplan–Meier curves demonstrated that PF-ILD was associated with poor survival in the FILD-UIP/PF-ILD, non-IPF-UIP/PF-ILD, and IPF/PF-ILD groups (log-rank test, all *p* < 0.05) (Figure 3). Moreover, FILD-UIP/PF-ILD, non-IPF-UIP/PF-ILD, and IPF/PF-ILD were similarly associated with poor survival rates (log-rank test, *p* = 0.42) (Figure 4).

## 4. Discussion

This study provided a comprehensive evaluation of the predictive factors and outcomes of PF-ILD in patients with FILD-UIP. Based on the INBUILD trial criteria, our data showed that non-IPF-UIP/PF-ILD occurred less frequently than IPF/PF-ILD (38.0% vs. 47.8%); however, this difference was not statistically significant. Nevertheless, non-IPF-UIP/PF-ILD had a significantly negative impact on overall survival, similar to IPF/PF-ILD. Wang et al. [3] reported that PF-ILD may develop in approximately 39.21% of patients with FILD, based on the 2020 INBUILD definition criteria. Takei et al. [28] reported that PF-ILD may develop in approximately 42.1% of patients with FILD overall, with 59.4% of IPF meeting the criteria compared to 26.6% of those with non-IPF FILD. In an analysis of 120 patients with non-IPF FILD, Goos et al. [29] reported that 68.3% met the INBUILD trial’s PF-ILD criteria. A large study conducted by pulmonologists, rheumatologists, and internists from multiple countries found that 18–32% of patients with non-IPF ILD had signs of progression and fibrosis [6]. Our results provide further evidence of the frequency of PF-ILD in patients with FILD-UIP.

In our study, baseline FVC% predicted was a significant independent predictor of PF-ILD in patients with FILD-UIP, non-IPF-UIP, and IPF. Lower pulmonary reserve at the time of UIP diagnosis, as measured by FVC%pred, increased the risk of progression. Impaired pulmonary physiology has been identified as a potential predictor of PF-ILD, and our data suggest that disease severity is closely associated with its incidence. Lower FVC is an established predictor of mortality in patients with IPF [30]. A retrospective cohort study found that a lower predicted FVC% at baseline increased the risk of progression in patients with RA-ILD and was further exacerbated by UIP [31]. Gimenez et al. [32] reported that a lower FVC% was predictive of mortality in patients with chronic HP, whereas Winstone et al. [33] conducted a systematic review and found that a lower FVC predicted both mortality and ILD progression in patients with SSc-ILD. These findings indicate that baseline disease severity is closely associated with the incidence of PF-ILD.

PH has been reported to complicate the course of several fibrotic lung diseases, including IPF, HP, and CTD-ILD, and its development is associated with decreased survival [25]. Across studies, the prevalence of PH among patients with IPF has ranged from 3% to 86%. Patients with IPF who develop PH may have pulmonary vasculopathy independent of ILD severity [34]. One prospective study found that right heart catheterization confirmed PH in 50% of patients with chronic HP [35]. Tyndall et al. [36] reported that the incidence of PH, based on echocardiography, was 26% in patients with SSc. Dawson et al. [37] reported that 21% of the patients had PH, whereas a retrospective study found that PH occurred in 33% of patients with idiopathic inflammatory myopathies (IIM). The predominant mechanisms of PH are hypoxemia and vascular remodeling [38]. Ahmad et al. [15] retrospectively analyzed a series of patients with IPAF and found that PH occurred in 22% of patients. In our study, PH was found in 25.4% of patients with IPF and 24.0% of patients with non-IPF-UIP. PH is an independent predictor of PF-ILD in patients with FILD-UIP, non-IPF-UIP, and IPF. Although SpO_2_ at baseline and post-exercise during the 6MWT were risk factors for PF-ILD, they were not independent predictors. This finding suggests that PH may be related to hypoxemia and vascular remodeling independent of fibrosis severity.

Our data showed that a lower KBILD score was an independent predictor of PF-ILD in patients with FILD-UIP and IPF. The KBILD is a brief QOL questionnaire developed for patients with ILDs. Fibrotic changes in FILD often result in decreased QOL. Health status is increasingly used in clinical practice to quantify symptom burden in patients with ILD. In their study, Lee et al. [9] reported that KBILD was significantly associated with mortality in 238 patients with IPF. Meanwhile, Sharp et al. [10] found that in 175 patients with ILD, KBILD was a dependent prognostic factor with a prognostic power similar to that of PFT and 6MWT. Assessing QOL is important for providing optimal prognostic information for patients with ILD. Our data support that patients with worse health status, as measured by KBILD, had a significantly increased risk of PF-ILD compared to those with better health status.

### Limitations

Our study has several limitations. First, its retrospective, single-center design means that longitudinal follow-up data were incomplete for some patients, which may affect the generalizability of the findings. Second, the definition of PF-ILD applied here is not a universally standardized one, and some potential cases may not have been fully captured within our hospital records system, limiting the comprehensiveness of the analysis. Third, the non-IPF UIP group itself is clinically heterogeneous, encompassing subtypes such as CTD-UIP, IPAF-UIP, and HP-UIP. Within this group, the relatively low number of outcome events (e.g., *n* = 12 deaths) constrained our ability to perform well-powered multivariable subgroup analyses. Furthermore, the lack of a standardized, systematic environmental exposure questionnaire means that potential triggers for HP could have been under-recognized. Implementing structured tools, such as the questionnaire used by Perlunk et al. [39], could improve detection in future studies. Fourth, basic treatment strategy is different between IPF and non-IPF-UIP groups. A proportion of non-IPF-UIP patients were already receiving antifibrotic therapy at baseline based on pre-enrollment clinical decisions. Although treatment type was not associated with progression outcome within groups, we cannot rule out unmeasured confounding by indication. While this introduces heterogeneity, it underscores the actual clinical context in which these diseases are managed. Further prospective studies involving larger populations are warranted, as patients with FILD-UIP were analyzed collectively. CTD-UIP and HP-UIP are the most frequent diagnoses in patients with non-IPF, and previously published data have shown that survival rates in these categories are lower than those in IPF [40,41,42]. Our study found no significant differences in the short-term outcomes of PF-ILD among the FILD-UIP subtypes. Future research should validate these findings.

## 5. Conclusions

The present study demonstrated that the development of PF-ILD significantly impacted overall survival in patients with FILD-UIP, non-IPF-UIP, and IPF. Patients with non-IPF-UIP who developed PF-ILD had poor outcomes comparable to those with IPF. This suggests that all forms of FILD-UIP are at risk for PF-ILD and exhibit similar outcomes to IPF-UIP/PF-ILD.

## Figures and Tables

**Table 1:** Comparison of patient characteristics of patients with FILD-UIP.

	IPF (*n* = 67)	Non-IPF-UIP (*n* = 50)	*p*-Value
Age at diagnosis, years	67.2 ± 8.5	63.1 ± 10.2	0.019
BMI(kg/m^2^)	24.5 ± 3.0	23.9 ± 2.9	0.339
Gender (male, %)	59 (88.1)	34 (68.0)	0.008
Smoking status (%)	45 (67.2)	26 (52.0)	0.097
Smoking pack-yrs	35.3 ± 17.1	32.2 ± 15.3	0.456
Pulmonary function at diagnosis			
FVC, % predicted	80.8 ± 20.7	80.4 ± 20.3	0.926
DLCO, % predicted	55.6 ± 21.6	57.1 ± 17.1	0.699
6MWT			
6MWD (m)	357.3 ± 177.4	351.6 ± 146.6	0.855
SpO_2_ baseline	91.0 ± 6.3	91.8 ± 5.6	0.516
SpO_2_ post-exercise	86.5 ± 8.9	84.7 ± 6.9	0.232
Pulse baseline	86.8 ± 15.3	85.5 ± 13.4	0.627
Pulse post-exercise	105.7 ± 18.1	109.8 ± 17.9	0.254
Non-IPF FILD-UIP subtypes (%)			
CTD-UIP	-	29 (24.8)	-
IPAF-UIP	-	14 (11.9)	-
HP-UIP	-	7 (6.0)	-
Treatment			
Antifibrotic treatment	59 (88.1)	17 (34.0)	<0.001
Steroid therapy	0 (0)	33 (66.0)	<0.001
Immunosuppressive therapy	0 (0)	23 (46.0)	<0.001
Comorbidities (*n*%)			
Lung cancer	6 (9.0)	2 (4.0)	0.464
PH (Echo-RVSP > 35 mmHg)	17 (25.4)	12 (24.0)	0.865
Mortality			
Deceased (%)	25 (37.3)	12 (24.0)	0.001
Average follow-up time/month	37.4 ± 14.6	41.1 ± 13.0	0.158

Values are presented as the mean ± SD or *n* (%). BMI, body mass index; CTD, connective tissue disease; DLCO, diffusion capacity of the lung for carbon monoxide; Echo-RVSP: echocardiography right ventricle systolic pressure; FILD, fibrosing interstitial lung disease; FVC, forced vital capacity; HP: hypersensitivity pneumonitis; 6MWT, 6-min walk test; 6MWD, 6-min walk distance; IPAF, interstitial pneumonia with autoimmune features; IPF, idiopathic pulmonary fibrosis; PH, pulmonary hypertension; UIP, usual interstitial pneumonia.

**Table 2:** Comparison of patient characteristics between the IPF and non-IPF-UIP groups stratified by PF-ILD status.

	IPF			Non-IPF-UIP		
	PF-ILD (*n* = 32)	Non-PF-ILD (*n* = 35)	*p*-Value	PF-ILD (*n* = 19)	Non-PF-ILD (*n* = 31)	*p*-Value
Age at diagnosis, years	67.2 ± 9.2	67.1 ± 7.9	0.971	66.4 ± 7.7	61.0 ± 11.0	0.071
BMI (kg/m^2^)	23.4 ± 3.0	25.4 ± 2.7	0.005	24.6 ± 2.7	23.5 ± 3.8	0.254
Gender (male, %)	28 (87.5)	31 (88.6)	1.000	15 (78.9)	19 (61.3)	0.194
Current smoker or ever smoked	23 (71.9)	22 (62.9)	0.432	11 (57.9)	15 (48.4)	0.514
Never smoked	9 (28.1)	13 (37.1)		8 (42.1)	16 (51.6)	
Smoking pack-yrs	33.3 ± 16.4	37.5 ± 17.9	0.436	31.7 ± 14.8	30.7 ± 15.3	0.181
Non-IPF FILD-UIP subtypes (%)						
CTD-UIP	-	-	-	10 (52.6)	19 (61.3)	0.547
IPAF-UIP	-	-	-	4 (21.1)	10 (32.3)	0.527
HP-UIP	-	-	-	5 (26.3)	2 (6.5)	0.089
Comorbidities (*n*%)						
Lung cancer	4 (12.5)	2 (5.7)	0.414	1 (5.3)	1 (3.2)	1.000
PH (Echo-RVSP > 35 mmHg)	15 (46.9)	2 (5.7)	<0.001	10 (52.6)	2 (6.5)	<0.001

Values are presented as the mean ± SD or *n* (%). BMI, body mass index; CTD, connective tissue disease; Echo-RVSP: echocardiography right ventricle systolic pressure; FILD, fibrosing interstitial lung disease; HP: hypersensitivity pneumonitis; IPAF, interstitial pneumonia with autoimmune features; IPF, idiopathic pulmonary fibrosis; PF-ILD, progressive fibrosing interstitial lung disease; PH, pulmonary hypertension; UIP, usual interstitial pneumonia.

**Table 3:** Comparison of QOL and functional parameters for IPF and non-IPF-UIP stratified by the PF-ILD status.

	IPF			Non-IPF-UIP		
	PF-ILD (*n* = 32)	Non-PF-ILD (*n* = 35)	*p*-Value	PF-ILD (*n* = 19)	Non-PF-ILD (*n* = 31)	*p*-Value
QOL						
KBILD	52.0 (45.0, 60.8)	70.0 (63.0, 82.0)	<0.001	55.0 (45.0, 69.0)	71.0 (63.0, 86.0)	0.011
LCQ	81.0 (71.0, 117.8)	104.0 (86.0, 121.0)	0.055	88.0 (79.0, 100.0)	97.0 (81.0, 109.0)	0.207
mMRC	3.0 (2.0, 3.0)	1.0 (1.0, 2.0)	<0.001	3.0 (2.0, 3.0)	2.0 (2.0, 2.0)	0.004
HADS	17.5 (8.3, 22.8)	9.0 (6.0, 17.0)	0.046	18.0 (9.0, 23.3)	12.0 (7.0, 20.0)	0.037
Physiology						
FVC, % predicted	71.3 ± 16.3	89.5 ± 20.6	<0.001	71.8 ± 17.9	85.7 ± 20.0	0.017
DLCO, % predicted	44.9 ± 17.2	63.1 ± 21.5	0.001	48.6 ± 18.0	60.8 ± 15.5	0.029
BGA						
PaO_2_/FiO_2_ ratio	282.4 ± 102.1	339.2 ± 69.0	0.012	287.1 ± 63.1	353.0 ± 98.7	0.039
6MWT						
6MWD (m)	284.8 ± 195.5	423.5 ± 129.6	0.001	266.1 ± 140.4	404.1 ± 125.8	0.001
SpO_2_ baseline	88.8 ± 7.3	93.0 ± 4.4	0.005	89.7 ± 4.3	93.0 ± 6.1	0.045
SpO_2_ post-exercise	83.9 ± 10.8	88.6 ± 6.6	0.038	80.8 ± 6.6	87.1 ± 5.9	0.001
Pulse baseline	89.0 ± 15.0	84.9 ± 15.6	0.280	86.9 ± 17.7	84.6 ± 10.1	0.558
Pulse post-exercise	109.0 ± 17.4	103.2 ± 18.5	0.225	115.9 ± 17.7	105.9 ± 17.3	0.07

Values are presented as the mean ± SD or median interquartile range. BGA, blood gas analysis; DLCO, diffusion capacity of the lung for carbon monoxide; FVC, forced vital capacity; HADS, Hospital Anxiety and Depression Scale; KBILD, King’s Brief Interstitial Lung Disease; LCQ, Leicester Cough Questionnaire; 6MWT, 6-min walk test; 6MWD, 6-min walk distance; mMRC, modified Medical Research Council; IPF, idiopathic pulmonary fibrosis; PF-ILD, progressive fibrosing interstitial lung disease; QOL: quality of life; UIP, usual interstitial pneumonia.

**Table 4:** Comparison of laboratory and treatment parameters for IPF and non-IPF-UIP stratified by the PF-ILD status.

	IPF			Non-IPF-UIP		
	PF-ILD (*n* = 32)	Non-PF-ILD (*n* = 35)	*p*-Value	PF-ILD (*n* = 19)	Non-PF-ILD (*n* = 31)	*p*-Value
Laboratory features						
LDH (U/L)	427.8 ± 296.1	284.3 ± 157.1	0.029	415.7 ± 250.5	335.8 ± 173.8	0.035
NLR	3.7 ± 2.5	3.2 ± 2.3	0.381	3.2 ± 2.5	2.6 ± 1.6	0.317
LMR	3.4 ± 1.9	3.5 ± 2.3	0.896	4.4 ± 2.6	4.0 ± 1.7	0.477
Albumin (g/L)	37.9 ± 6.0	40.2 ± 4.7	0.081	36.3 ± 4.7	37.1 ± 4.6	0.584
Total cholesterol (mmol/L)	4.2 ± 1.0	3.9 ± 0.7	0.125	4.2 ± 1.1	3.8 ± 0.9	0.131
Treatment						
Antifibrotic treatment	30 (93.8)	29 (82.9)	0.262	6 (31.6)	11 (35.5)	0.513
Steroid therapy	0	0	1.000	10 (52.6)	23 (69.7)	0.137
Immunosuppressive therapy	0	0	1.000	7 (36.8)	16 (48.5)	0.387
Mortality						
Deceased (%)	22 (68.8)	3 (8.6)	<0.001	10 (52.6)	2 (6.5)	<0.001
Average follow-up time/month	34.6 ± 18.2	40.0 ± 9.6	0.130	31.3 ± 14.1	42.1 ± 14.8	0.043

Values are presented as the mean ± SD or *n* (%). IPF, idiopathic pulmonary fibrosis; LDH: lactic dehydrogenase; LMR: lymphocyte-to-monocyte ratio; NLR: neutrophil-to-lymphocyte ratio; PF-ILD, progressive fibrosing interstitial lung disease; UIP, usual interstitial pneumonia.

**Table 5:** Factors associated with PF-ILD.

**(A) All FILD-UIP (** ***n*** ** = 117; PF-ILD,** ** * n*** ** = 51)**				
**Covariate**	**Univariable OR (95% CI)**	***p*** **-Value**	**Multivariable OR (95% CI)**	***p*** **-Value**
PH (Echo-RVSP > 35 mmHg)	15.750 (4.974–49.875)	<0.001	6.056 (1.664–22.047)	0.006
KBILD	9.735 (4.147–22.849)	<0.001	4.529 (1.592–12.884)	0.005
mMRC	5.736 (2.512–13.101)	<0.001		
HADS	2.103 (0.996–4.441)	0.051		
FVC% pred	4.787 (2.144–10.690)	<0.001	3.240 (1.071–9.800)	0.037
DLCO% pred	6.352 (2.756–14.640)	<0.001		
PaO_2_/FiO_2_ ratio	5.228 (2.317–11.796)	<0.001		
6MWD (m)	5.091 (2.173–11.929)	<0.001		
SpO_2_ baseline	7.000 (3.082–15.898)	<0.001		
SpO_2_ post-exercise	4.055 (1.842–8.926)	0.001		
LDH (U/L)	1.939 (0.795–4.726)	0.145		
**(B) Non-IPF-UIP (** ***n*** ** = 50; PF-ILD,** ***n*** ** = 19)**				
**Covariate**	**Univariable OR (95% CI)**	***p*** **-Value**	**Multivariable OR (95% CI)**	***p*** **-Value**
PH (Echo-RVSP > 35 mmHg)	18.750 (3.402–103.335)	0.001	11.123 (1.833–67.491)	0.009
KBILD	4.464 (1.277–15.608)	0.019		
mMRC	5.612 (1.584–19.886)	0.008		
HADS	1.781 (0.550–5.766)	0.336		
FVC% pred	8.667 (2.310–32.516)	0.001	4.762 (1.074–21.103)	0.04
DLCO% pred	4.333 (1.235–15.206)	0.022		
PaO_2_/FiO_2_ ratio	4.320 (1.140–16.371)	0.031		
6MWD (m)	4.333 (1.203–15.605)	0.025		
SpO_2_ baseline	10.500 (2.670–41.292)	0.001		
SpO_2_ post-exercise	11.692 (2.290–59.705)	0.003		
LDH (U/L)	1.154 (0.251–5.300)	0.854		
**(C) IPF (** ***n*** ** = 67; PF-ILD,** ***n*** ** = 32)**				
**Covariate**	**Univariable OR (95% CI)**	***p*** **-Value**	**Multivariable OR (95% CI)**	***p*** **-Value**
PH (Echo-RVSP > 35 mmHg)	14.559 (2.977–71.193)	0.001	8.022 (1.104–58.282)	0.04
KBILD	17.333 (5.148–58.364)	<0.001	16.297 (3.854–68.918)	<0.001
mMRC	6.214 (2.022–19.098)	0.001		
HADS	2.889 (1.034–8.068)	0.043		
FVC% pred	11.455 (3.467–37.848)	<0.001	5.478 (1.195–25.127)	0.029
DLCO% pred	8.306 (2.685–25.691)	<0.001		
PaO_2_/FiO_2_ ratio	5.515 (1.926–15.792)	0.001		
6MWD (m)	6.000 (1.856–19.395)	0.003		
SpO_2_ baseline	5.500 (1.930–15.673)	0.001		
SpO_2_ post-exercise	2.821 (1.047–7.599)	0.04		
LDH (U/L)	2.889 (0.946–8.818)	0.062		

DLCO, diffusion capacity of the lung for carbon monoxide; Echo-RVSP, echocardiography right ventricle systolic pressure; FILD, fibrosing interstitial lung disease; FVC, forced vital capacity; HADS, Hospital Anxiety and Depression Scale; IPF, idiopathic pulmonary fibrosis; KBILD, King’s Brief Interstitial Lung Disease; LDH, lactic dehydrogenase; 6MWD, 6-min walk distance; mMRC, modified Medical Research Council; PF-ILD, progressive fibrosing interstitial lung disease; PH, pulmonary hypertension; UIP, usual interstitial pneumonia.

## Data Availability

The data that support the findings of this study are available on reasonable request from the corresponding author. However, the data are not publicly available due to privacy or ethical restrictions.

## Supplementary Materials

The following supporting information can be downloaded at: https://www.mdpi.com/article/10.3390/medicina62010206/s1, Table S1. Comparison of patient characteristics of patients with different Non-IPF-UIP. Table S2. Factors associated with overall mortality.
