# Supplementary material for: Predictors of Progressive Fibrosing Interstitial Lung Diseases and Survival in Fibrosing Interstitial Lung Disease-Related Usual Interstitial Pneumonia
Source: Medicina (Kaunas). 2026 Jan 19;62(1):206. doi: 10.3390/medicina62010206 (PMC12843998; doi:10.3390/medicina62010206)
Supplement: Supplementary file 1 [file medicina-62-00206-s001.zip › medicina-4041015-supplementary.pdf]

Table S1. Comparison of patient characteristics of patients with different Non-IPF-UIP.

	CTD-UIP (n=29)	IPAF-UIP (n=14)	HP-UIP (n=7)	*p*-value
Age at diagnosis, years	61.2±12.0	66.4.1±5.3	64.3±8.0	0.280
BMI(kg/m2)	23.6±.3.6	24.4±3.4	24.0±3.2	0.785
Gender (male, %)	17 (58.6)	13 (92,9)	4 (57.1)	0.008
Smoking status (%)	11 (37.9)	12 (78.6)	3 (42.9)	0.097
Smoking pack-yrs	33.5±12.9	35.0±16.3	38.0±26.9	0.887
Pulmonary function at diagnosis				
FVC, % predicted	77.6±21.9	77.2±18.9	76.6±4.6	0.246
DLCO, % predicted	55.5±17.6	55.1±14.9	66.4±18.6	0.300
6MWT				
6MWD (m)	354.3±150.7	341.7±155.8	360.1±129.5	0.954
SpO2 baseline	92.8±4.1	89.4±8.1	92.3±4.7	0.187
SpO2 post-exercise	85.0±6.0	84.8±8.0	83.6±6.7	0.881
Pulse baseline	88.8±12.8	85.2±13..0	82.1±8.3	0.481
Pulse post-exercise	115.2±18.6	101.1±16.4	105.1±11.5	0.057
Treatment				
Antifibrotic treatment	7 (24.1)	7 (50)	3 (42.9)	0.212
Steroid therapy	22(75.9)	6 (42,9)	5 (71.4)	0.096
Immunosuppressive therapy	18 (62.1)	5 (35.7)	0 (0)	0.008
Comorbidities (n%)				
Lung cancer	2 (6.9)	0 (0)	0 (0)	0.470
PH (Echo-RVSP > 35 mm	8 (27.6)	3 (21.4)	1(14.2)	0.734

Hg)				
Average follow-up time/month	42.9±12.7	34.6±11.6	46.4±12.5	0.068

Table S2. Factors associated with overall mortality.

(A) All FILD-UIP (IPF/Non-IPF-UIP) (n=117; death, n=37)				
Covariate	Univariable HR (95% CI)	p-value	Multivariable HR (95% CI)	p-value
PF-ILD	8.848 (3.662-21.375)	<0.001	6.324 (2.442-16.379)	<0.001
PH (Echo-RVSP > 35 mmHg)	4.941 (2.503-9.754)	<0.001	0.459 (0.220-0.959)	0.038
KBILD	5.657 (2.467-12.976)	<0.001		
mMRC	2.908 (1.487-5.688)	0.002		
HADS	1.192 (0.612-2.322)	0.606		
FVC% pred	3.724 (1.823-7.607)	<0.001		
DLCO% pred	3.293 (1.494-7.261)	0.003		
PaO2/FiO2 ratio	1.846 (0.947-3.601)	0.072		
6MWD (m)	2.184 (1.119-4.262)	0.022		
SpO2 baseline	2.131 (1.081-4.198)	0.029		

SpO2 post-exercise	2.810 (1.373-5.754)	0.005		
LDH (U/L)	1.794 (0.775-4.149)	0.172		
(B) Non-IPF-UIP (n=50; death, n=12)				
Covariate	Univariable HR (95% CI)	p-value	Multivariable HR (95% CI)	p-value
PF-ILD	12.887 (1.581-105.018)	0.017	14.270 (1.640-124.150)	0.002
PH (Echo-RVSP > 35 mmHg)	5.430 (1.294-22.783)	0.021		
KBILD	14.949 (1.862-120.031)	0.011		
mMRC	6.666 (1.371-32.417)	0.019		
HADS	6.884 (0.847-55.977)	0.071		
FVC% pred	4.093 (0.822-20.372)	0.085	1.218 (0.301-4.935)	0.782
DLCO% pred	2.543 (0.512-12.621)	0.254		
PaO2/FiO2 ratio	1.705 (0.407-7.148)	0.465		
6MWD (m)	9.219 (1.833-46.379)	0.007		
SpO2 baseline	2.923	0.132		

	(0.724-11.799)			
SpO2 post-exercise	4.944 (0.608-40.213)	0.135		
LDH (U/L)	1.690 (0.208-13.752)	0.624		
(C) IPF (n=67; death, n=25)				
Covariate	Univariable HR (95% CI)	p-value	Multivariable HR (95% CI)	p-value
PF-ILD	5.058 (1.733-14.767)	0.003	4.939 (1.313-18.577)	0.018
PH (Echo-RVSP > 35 mm Hg)	3.248 (1.444-7.304)	0.004		
KBILD	4.029 (1.509-10.757)	0.005		
mMRC	1.900 (0.866-4.171)	0.109		
HADS	0.780 (0.342-1.776)	0.553		
FVC% pred	4.624 (1.924-11.112)	0.001	0.354 (0.133-0.942)	0.038
DLCO% pred	3.438 (1.283-9.212)	0.014		
PaO2/FiO2 ratio	1.415 (0.640-3.131)	0.391		
6MWD (m)	1.375 (0.619-3.055)	0.434		
SpO2 baseline	1.450	0.364		

	(0.650-3.234)	
SpO2 post-exercise	2.444<br>(1.076-5.552)	0.033
LDH (U/L)	0.755(0.755-4.784)	1.901
